# Chronic Levodopa Administration Followed by a Washout Period Increased Number and Induced Phenotypic Changes in Striatal Dopaminergic Cells in MPTP-Monkeys

**DOI:** 10.1371/journal.pone.0050842

**Published:** 2012-11-30

**Authors:** Carla DiCaudo, Mario Riverol, Iñaki-Carril Mundiñano, Cristina Ordoñez, María Hernández, Irene Marcilla, Maria-Rosario Luquin

**Affiliations:** Laboratory of Regenerative Therapy and Department of Neurology, Division of Neuroscience, University of Navarra, Pamplona, Spain; University of Nebraska Medical Center, United States of America

## Abstract

In addition to the medium spiny neurons the mammalian striatum contains a small population of GABAergic interneurons that are immunoreactive for tyrosine hydroxylase (TH), which dramatically increases after lesions to the nigrostriatal pathway and striatal delivery of neurotrophic factors. The regulatory effect of levodopa (L-Dopa) on the number and phenotype of these cells is less well understood. Eleven macaques (Macaca fascicularis) were included. Group I (n = 4) received 1-methyl-4-phenyl-1,2,3,6 tetrahydropyridine (MPTP) and L-Dopa; Group II (n = 4) was treated with MPTP plus vehicle and Group III (n = 3) consist of intact animals (control group). L-Dopa and vehicle were given for 1 year and animals sacrificed 6 months later. Immunohistochemistry against TH was used to identify striatal and nigral dopaminergic cells. Double and triple labeling immunofluorescence was performed to detect the neurochemical characteristics of the striatal TH-ir cells using antibodies against: TH, anti-glutamate decarboxylase (GAD_67_) anti-calretinin (CR) anti-dopa decarboxylase (DDC) and anti-dopamine and cyclic AMP-regulated phosphoprotein (DARPP-32). The greatest density of TH-ir striatal cells was detected in the striatum of the L-Dopa treated monkeys and particularly in its associative territory. None of the striatal TH-ir cell expressed DARPP-32 indicating they are interneurons. The percentages of TH-ir cells that expressed GAD67 and DDC was approximately 50%. Interestingly, we found that in the L-Dopa group the number of TH/CR expressing cells was significantly reduced. We conclude that chronic L-Dopa administration produced a long-lasting increase in the number of TH-ir cells, even after a washout period of 6 months. L-Dopa also modified the phenotype of these cells with a significant reduction of the TH/CR phenotype in favor of an increased number of TH/GAD cells that do not express CR. We suggest that the increased number of striatal TH-ir cells might be involved in the development of aberrant striatal circuits and the appearance of L-Dopa induced dyskinesias.

## Introduction

The striatum is the main afferent structure of the basal ganglia. It is primarily composed of GABAergic spiny projection neurons that make up approximately 95% of all the striatal neurons in rodents. The proportion is significantly lower in higher vertebrates, especially primates (77%) [1]. The cholinergic neurons make up only 0.5–1% of the neurons. The remaining neurons, comprising approximately 3–4% of the total number of neurons in the rodent striatum, are made up of aspiny GABAergic interneurons [2], [3], which have been classified according to their morphological and neurochemical characteristics into 3 different subtypes. A small population of these GABAergic interneurons is immunoreactive for tyrosine hydroxylase (TH-ir), the rate-limiting enzyme in catecholamine synthesis. These TH-ir cells have been preferentially found in the anterior striatum of several species including rat, mouse [4]–[6], monkey [7]–[11] and human [11]–[13]. These cells seem to express the machinery required for the synthesis, storage and release of dopamine and the orphan nuclear receptor Nurr1, which is essential for the development of the dopaminergic phenotype [14]. Although Ibañez-Sandoval et al. [15] have recently demonstrated in mouse that these TH-ir cells are well integrated into the functional synaptic organization of the neostriatum, and the integration of these neurons in the striatal microcircuitry has also been reported by electron microscopy in monkeys [9], their functional significance is still under debate. Interestingly, the number of the TH-ir striatal dopaminergic cells markedly increases after the lesion of nigrostriatal pathway both in rodents and primates, suggesting that they might act as a local source of dopamine (DA) [8], [12], [13], [16].

On the other hand, in PD patients and in 1-methyl-4-phenyl-1,2,3,6-tetrahydropyridine (MPTP)-monkeys, L-Dopa administration seems to reverse the numerical increase in striatal dopaminergic cells created by MPTP exposure [11], [17]. In fact, the number of striatal dopaminergic cells is much lower in L-Dopa MPTP-monkeys and PD patients treated with L-Dopa than in non-treated parkinsonian monkeys and age-matched controls, indicating that the striatal DA content is a critical regulatory factor of the number of striatal dopaminergic cells [18]. However, in the previous report, MPTP-monkeys received L-Dopa for a short period of time (1 month) and they were sacrificed immediately after the interruption of L-Dopa administration. Thus the reduced number of striatal TH-ir cells they reported might merely reflect an acute pharmacological effect related to L-Dopa administration.

In the current study, we assessed the impact of chronic L-Dopa administration on striatal TH-ir cells after a washout period of 6 months. We examined whether chronic L-Dopa treatment modifies the number, distribution and phenotype of striatal TH-ir neurons in monkeys with mild parkinsonism. We have paid attention on the possibility that L-Dopa can modify a particular phenotype of this cell population.

## Materials and Methods

### Animals and Study Design

A total of 11 adult (4–5 years old), male monkeys (*Macaca fascicularis*) weighting 3–5 kg and aged 4–5 years, were included in study. Animals were housed in an animal room under standard conditions of air exchange (16 l/min), humidity (50%), and light/night cycles (8 a.m. to 8 p.m.), and were fed fresh fruit and commercial pellets, with free access to water. Their health was monitored by the attending veterinarian consistent with the recommendations of the Weatherall Report. The animals were euthanized following deep anesthesia, and all efforts were made to minimize suffering. Experimental protocol was in accordance with the European Communities Council Directive of 24 November 1986 (86/609/EEC) and it was approved by the Ethics Committee for Animal Experimentation of the University of Navarra (Permit Number: 006/06).

**Figure 1:**
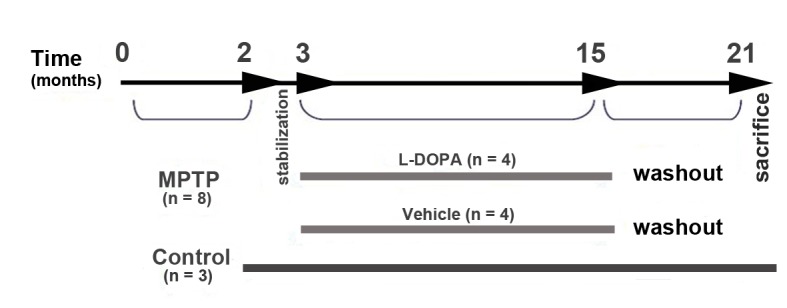
Experimental study design, treatments and group assignments.

Eight of the 11 monkeys received one weekly (for eight consecutive weeks), intravenous injection of MPTP (Sigma) dissolved in sterile saline at a dose of 0.25–0.5 mg/kg up to a cumulative MPTP dose of 2 mg/kg. We used this schedule since we planned to induce a dopaminergic cell loss no greater than 50%. The degree of nigral cell loss was monitored *in vivo* by changes in the uptake of F-Dopa PET scan. When animals exhibited a 50% reduction of the striatal F-Dopa uptake they were blindly allocated to receive L-Dopa or vehicle. Motor deficits induced by MPTP were assessed according to a non-human primate disability rating scale, which independently scores from 0 (normal) to 3 (maximum disability) parkinsonian features such as tremor (intensity and duration), balance, feeding and freezing; from 0 (normal) to 4 (maximum disability) bradykinesia and posture, and from 0 (normal) to 5 (maximum disability) the reduction in spontaneous activity, thus giving a total maximum score of 28 [19]. Baseline parkinsonian scores were collected and video recorded for at least 3 consecutive days during the week that preceded treatment with L-Dopa or vehicle and the scores were similar in both groups of MPTP-monkeys. We did not exclude any animal because all of them developed mild but stable parkinsonian symptoms 4 weeks after concluding MPTP treatment. Four MPTP-monkeys were started on a chronic daily oral treatment with L-Dopa/benserazide (Madopar®). In our experience the dose of L-dopa given (30 mg/kg/day) is able to reverses parkinsonism in the majority of MPTP-monkeys without provoking motor hyperactivity. L-Dopa dose was adjusted every month according to the weight of the animals. The total daily L-Dopa dose was then divided into 3 equal doses and administered dissolved in orange juice in the morning, noon and afternoon. Only during weekends animals received the total L-Dopa dose in a single dose. The other 4 MPTP-monkeys received the same volume of fruit juice (vehicle group). L-Dopa and vehicle treatments were maintained for 12 months and then discontinued. Six months after interrupting L-Dopa and vehicle treatments (18 months after the last MPTP injection), animals were euthanized. We established a washout period of 6 months since these animals had previously participated in another study in which the main objective was determinate the changes in the striatal ^18^-F-DOPA uptake assessed by PET scans and their reversibility after a washout period. The remaining three intact animals were not given MPTP or L-Dopa and served as controls (Control group). (Figure 1).

### Animal Perfusion and Tissue Preparation

Six months after discontinuing L-Dopa or vehicle treatment, animals were sacrificed. After deep sedation with a mixture of ketamine (10 mg/kg) and midazolam (1 mg/kg), animals were transcardially perfused with 0.01 M phosphate buffer saline (PBS) and 4% paraformaldehyde (PFA, Sigma) in 0.01 M PBS. Brains were immediately removed, blocked and post-fixated two days in 4% PFA. They were then cryoprotected in a 30% sucrose solution in 0.01 M PBS until processing. Brains were sliced into 40 µm-thick coronal sections along the rostral axis with a freezing microtome (Leica, Germany) and collected in 0.125 M PBS containing 2% dimethylsulphoxide (Sigma), 20% glycerin (Panreac) and 0.05% sodium azide and were stored at −20°C until their subsequent analysis. Free-floating immunohistochemistry and immunofluorescence techniques were used for histological analysis of the brain tissue sections.

### Immunohistochemistry

Tissue sections containing either the anterior or posterior striatum were washed in bi-distilled water and 0.01 M PBS to remove the cryoprotectant solution and incubated in PBS with 0.02% hydrogen peroxide (H2O2) for endogenous peroxidase inhibition. After that, they were incubated in 5% normal goat serum with 0.2% Triton X-100 (Sigma) for 30 min and then incubated overnight in the same solution containing a primary antibody to tyrosine hydroxylase (TH) (mouse monoclonal antibody, dilution 1∶1000; Millipore, CA), rinsed in 0.01 M PBS and incubated for 30 min in 0.01 M PBS containing the corresponding secondary antibody (biotinylated goat anti-mouse, 1∶200; Dako). Subsequently, they were incubated with the Vector avidin–biotin complex (1∶200, Vectastain Elite ABC kit, Vector Laboratories) for 30 min. Staining for peroxidase was performed in DAB substrate kit (Vector Laboratories, USA). Finally, the sections were rinsed in bidistilled water and 0.01 M PBS, mounted on gelatin-coated slides and air-dried. The next day, sections were Nissl counterstained and coverslipped using DPX (BDH). Immunoreactivity for TH in tissue sections of substantia nigra (SN) was tested following the same protocol.

### Immunofluorescence

Double-labeling immunofluorescence (IF) was performed to detect the neurochemical characteristics of the striatal TH-ir. Analysis was performed on 3 pre-commissural striatal sections of each animal. The following antibodies against different markers of striatal and DAergic neurons were used to characterize these cells: anti-glutamate decarboxylase (GAD_67_) (Mouse monoclonal antibody, dilution 1∶1000; Millipore, CA), anti-calretinin (CR) (Goat polyclonal antibody, dilution 1∶1000; Millipore, CA), anti-dopa decarboxylase (DDC) (Rabbit polyclonal antibody, dilution 1∶500, Millipore, CA) and anti-dopamine and cyclic AMP-regulated phosphoprotein (DARPP-32) (Rabbit polyclonal antibody, dilution 1∶100; Cell Signaling). Coronal striatal tissue sections were rinsed and permeabilized following the protocol used for peroxidase immunohistochemistry (see above). Subsequently, they were incubated overnight at 4°C in a solution containing a mixture of primary anti-TH (monoclonal or rabbit polyclonal, dilution 1∶1000; Millipore, CA) with the following antibodies: anti-DDC, anti-GAD67, anti-DARPP-32 or anti-CR. After rinsing with 0.01 M PBS, sections were incubated for 2 h in 0.01 M PBS containing normal donkey/goat serum (1∶20) and the corresponding combination of secondary antibodies coupled to fluorescent markers, Alexa Fluor 568 or Alexa Fluor 488 (Molecular Probes). Finally, sections were counterstained with a nucleic acid stain (TO-PRO-3 iodide, Molecular Probes, Netherlands) and coverslipped with mounting medium (Immu-mount, Thermo-Shandon).

To better characterize the striatal TH-ir cell population, triple-labeling immunofluorescence (IF) was performed on 3 pre-commissural striatal sections of two animals of each experimental group. In addition to TH antibody we used also antibodies against CR and GAD67, markers of striatal interneurons. Triple immunofluorescence was performed as follows. Striatal sections were incubated overnight at 4°C and the corresponding secondary antibodies (Alexa Fluor 633 and Alexa Fluor 488 respectively) used as described above. Subsequently, tissue sections were incubated with primary anti-TH polyclonal antibody and then with the secondary antibody (Alexa Fluor 555). At the end of the labeling process the sections were coverslipped with mounting medium.

### TH-ir Cell Counting and Phenotypic Characterization

TH-ir cell counting was performed in the striatum and in the substantia nigra pars compacta (SNpc) of all animals. For cell counting in the SNpc we used thirteen sections of the right SNpc per animal taken at the same antero-posterior level and was performed by an investigator aware of the assigned treatment. The estimation of the total number of TH-ir cells was carried out using the optical fractionator principle. Stereological analysis was performed using an Olympus BX-51 microscope with Olympus CAST system version 2.0 (Olympus, Denmark A/S, Albertslund, Denmark). The analyzed regions were outlined under a low magnification (UPlanApo 4×/0.16, Olympus) objective at all levels in the rostrocaudal axis. From a random start position a counting frame was superimposed on the image and neurons were systematically sampled using a 60× lens (Plan Apo N 60×/1.42 oil, Olympus) with the nucleolus used as the sampling unit. The sampling frequency was chosen by adjusting the xy-axis step length so that up to 200 cells were counted in each specimen. The percentage of neuronal loss in the SNpc was calculated with respect the value obtained in the intact animals (Table 1).

**Table 1:** General characteristics of the animals and TH-ir cell counting in the striatum and substantia nigra.

Group	Cumulative MPTP dose (mg/k)	Parkinsonian disability score (0–28)	Dopamine cell loss in the SN (%)	TH-ir cell density pre-commisural striatum (TH-ir cells/100 mm2)	TH-ir cell density post-commisural striatum (TH-ir cells/100 mm2)
Control (n = 3)	–	0	0	43.1±8.7	41.7±9.8
Vehicle (n = 4)	2	5±1.41	40.2±6.4	75.7±5.2*	52.1±5.2
L-Dopa (n = 4)	2	5.75±1.47	43.7±20.3	95.6±2.8*#	48.3±7.7

* : *P*≤0.05 compared to vehicle or L-Dopa with control group.

#: *P*≤0.05 compared to L-Dopa with vehicle group. The data show the mean and its corresponding SEM.

In the striatum, cell counting was undertaken in the right striatum (caudate and putamen) with bright-field microscopy using a 20× objective lens (Olympus BX51, Denmark) on six anatomically matched brain tissue sections taken at different levels of the pre-commissural striatum (AP: from +2 to +5 mm according to the atlas of Martin-Bowden [20] and in four tissue sections of the post-commissural striatum (from 0 to −5 mm). For each striatal section the TH-ir cell density (number of TH-ir neurons per 100 mm2) was calculated by dividing the total number of TH-ir cells by the area of the striatum surveyed. Then, TH-ir cell density was independently calculated for the anterior (first three sections) and posterior (last three sections) pre-commissural striatum. The mean value of each anatomical level (anterior and posterior) was used for the statistical analysis.

In order to characterize the phenotype of the striatal TH-ir cells, double and triple immunofluorescence techniques with different antibodies were performed in at least three non-consecutive sections of the pre-commissural striatum (levels +5, +3–4 and +2) in all animals for the double colocalization and in two animals per group for the triple colocalization. Double and triple-labeled cells were detected in confocal images by using a laser scanning microscope 510, equipped with three lasers (LSM 510/Meta; Zeiss, Germany). We counted the number of double and triple-labeled neurons in each tissue section. Then, the percentage of labeled cells with respect to the total number of striatal TH-ir cells was calculated in each tissue section. Control sections processed without primary antibodies showed a lack of immunolabeling. Striatal TH-ir cell counting and phenotypic characterization of the cells were blindly undertaken.

### Statistical Analysis

Multiple comparisons were performed in order to estimate the overall significance of parkinsonian disability, nigral dopamine cells loss and striatal TH-ir cell density, by using a non-parametric Kruskal-Wallis test followed by Mann-Whitney U-test (2–2).

For colocalization studies the number of single, double and triple-labeled striatal TH-ir neurons was averaged for each section and One way analysis of variance (ANOVA) with repeated measures followed by the Tukey test was used to estimate overall significance. Probability (p) values less than or equal to 0.05 were considered to be statistically significant. SPSS 15.0 software was employed.

## Results

### Behavioral Manifestations and Loss of Dopaminergic Cells in SNpc

MPTP administration elicited mild motor deficits in all animals consisting of bradykinesia, postural instability and tremor (Table 1). None of the MPTP intoxicated animal required L-Dopa treatment during the induction of parkinsonism. L-Dopa and vehicle treatment was initiated 3 weeks after concluding MPTP administration. Very mild dyskinesias appeared after two months of L-Dopa treatment in only one animal and consisted of dystonic or choreic involuntary movement in the legs. The mild intensity of the abnormal movements did not permit their quantification.

A marked and significant loss of dopaminergic neurons was observed in the SNpc of all MPTP-monkeys (40% cell loss approximately). No significant differences were observed between L-Dopa and vehicle groups, indicating that L-Dopa does not alter the survival of dopaminergic cells in the SNpc (Table 1).

### L-Dopa Further Increased the Number of Striatal TH-ir Cells Induced by MPTP

**Figure 2:**
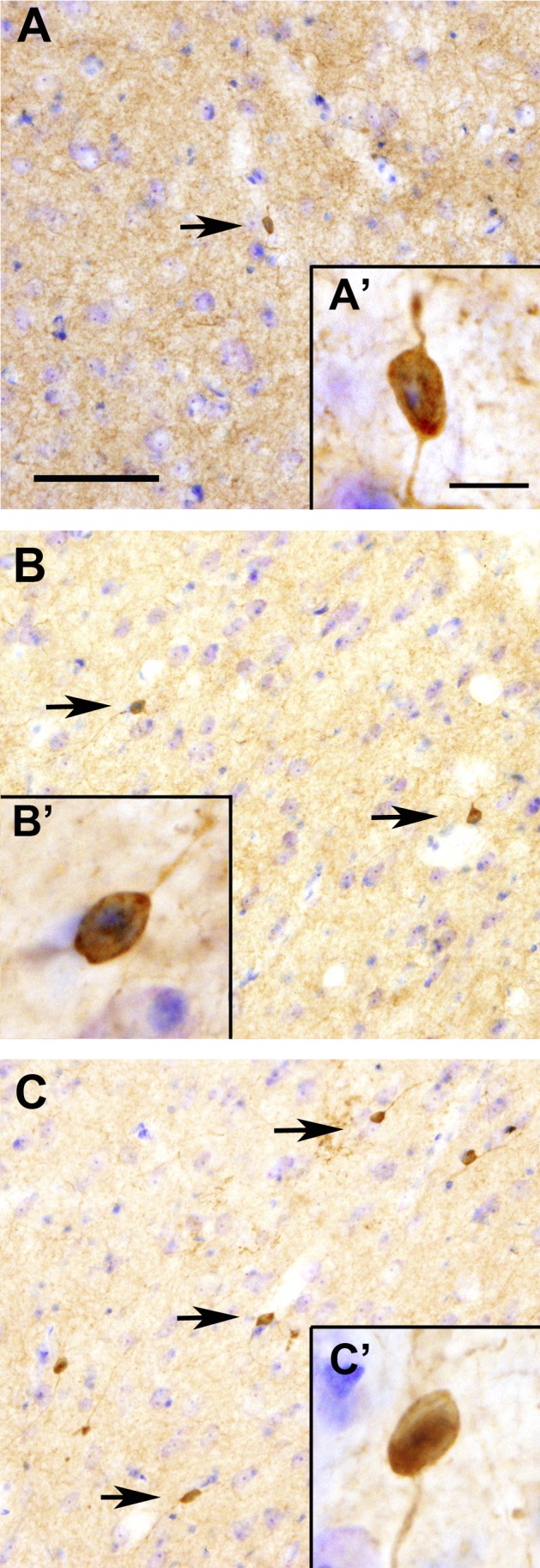
Striatal coronal sections immunostained with TH and counterstained with Nissl showing the morphology of the striatal dopaminergic cells. Control monkey (A) vehicle-treated monkey (B) and L-Dopa-treated monkey (C). Scale bar, 100 µm (A-C) and 20 µm (A’–C’).

In order to guarantee the cell nature of all striatal TH-ir labeled structures we only quantified those with a Nissl-labeled nucleus and at least one neurite. TH-ir cells were found within the striatum of every experimental group. In all groups of animals, the majority of TH-ir cells were aspiny and bipolar neurons with a round or oval perikaryon as previously described [8], [21] (Figure 2).

**Figure 3:**
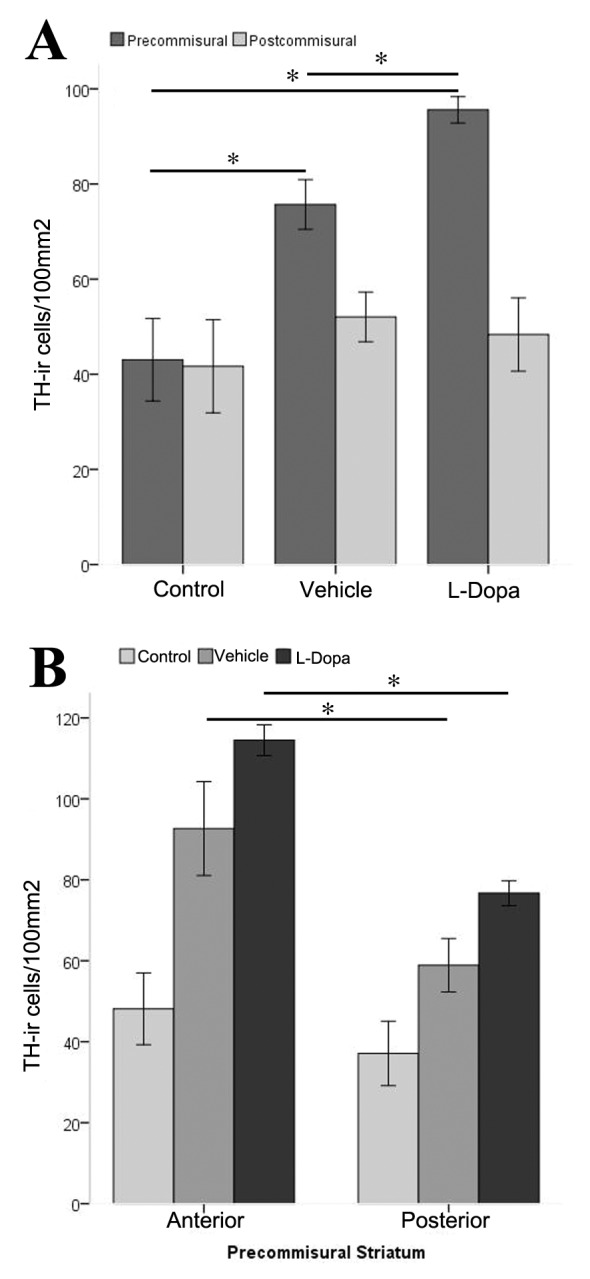
Striatal TH-ir cell distribution. (A) Mean of density of TH- ir cells in the pre- commissural and post-commissural striatum of control (n = 3), vehicle (n = 4) and L-Dopa (n = 4) groups. Mann and Whitney U: * = p<0.05. (B) Mean density of TH-ir cells in the anterior and posterior parts of pre-commissural striatum of control (n = 3), vehicle (n = 4) and L-Dopa (n = 4) groups. Mann and Whitney U: * = p<0.05.

Pre and post-commissural striatal sections were analyzed separately (Figure 3A). In the pre-commissural striatum the density of TH-ir neurons of the L-Dopa treated monkeys was increased twofold with respect to control animals (p = 0.034). Thus, in the control monkeys, the average density of TH-ir cells in the precommissural striatum was 43.1±8.6 cells/100 mm2, while in the L-Dopa group it increased to 95.6±2.8. Similarly, the density of TH-ir cells was greater in the L-Dopa than in the vehicle group (p = 0.021), which in turn was also greater than the striatal TH-ir cell density of the intact animals (p = 0.034). On the other hand, in the post-commissural striatum the density of TH-ir cells was similar in all experimental groups (control: 41.7±9.8, vehicle: 52.1±5.2, L-Dopa: 48.3±7.7; p = 0.89). These results indicate that L-Dopa and MPTP preferentially increased the number of TH-ir in the pre-commissural striatum.

### Regional Distribution of Striatal TH-ir Cells in the Pre-commissural Striatum

**Figure 4:**
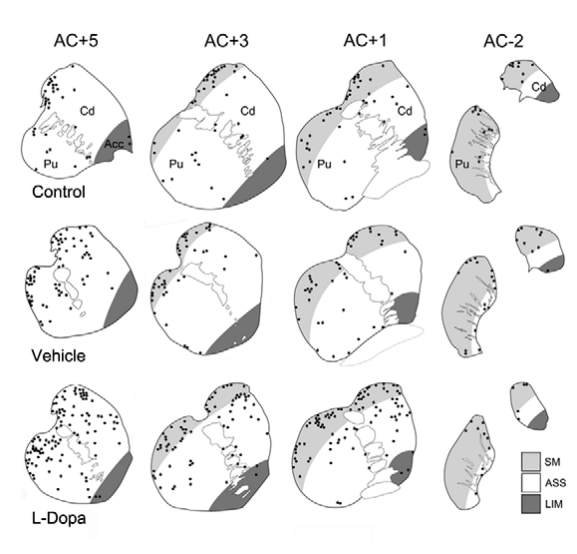
Graphic representation of the distribution of striatal TH-ir neurons in each experimental group. Note that L-Dopa group has the highest number of TH-ir cells, particularly in the more rostral areas. The delineation of territory boundaries based on anatomical assessments is also showed. Sensorimotor (SM), Associative (ASS) and Limbic (Lim) territories of the caudate (Cd), Putamen (Pu) and part of nucleus accumbens (Acc) are included. The distances from the anterior commissure (AC) are indicated.

Since the striatal dopaminergic cells are particularly profuse in the pre-commissural striatum we studied the distribution and morphology of the striatal TH-ir cells in this striatal region using bright-field microscope images. As we describe above, TH-ir neurons displayed a particular pattern of distribution, being especially abundant in the rostro-dorsal region of the caudate nucleus and putamen [8] (Figure 4).

Subsequently, we divided the pre-commissural striatum into anterior and posterior parts and 3 striatal tissue sections of each area were separately analyzed for TH-ir cell counting. We found that in the control group the number of TH-ir cells was similar in the anterior (48,1±8.8) and posterior (37,1±7,9) part of the pre-commissural striatum (p = 0.41) while in vehicle (anterior: 92,6±11,6 and posterior: 58,9±6,6) and L-Dopa groups (anterior: 114,5±3,8 and posterior: 76,7±3,1) the number of TH-ir cells was significantly higher (p = 0.045 and p<0.01 respectively) in the more anterior part of the pre-commissural striatum (caudate and putamen) (Figure 3B).

### Neurochemical Characteristics of the Striatal TH-ir Cells

To better characterize the phenotype of the striatal TH-ir cells, we first performed double-IF techniques with antibodies against DAergic markers such as DDC and some common markers of striatal neurons such as GAD67, CR and DARPP-32. As degenerating swollen DAergic axons might be mistaken for bipolar cell bodies, we firstly verified that all the TH-ir structures also expressed the nuclear marker TO-PRO3 [22].

**Figure 5:**
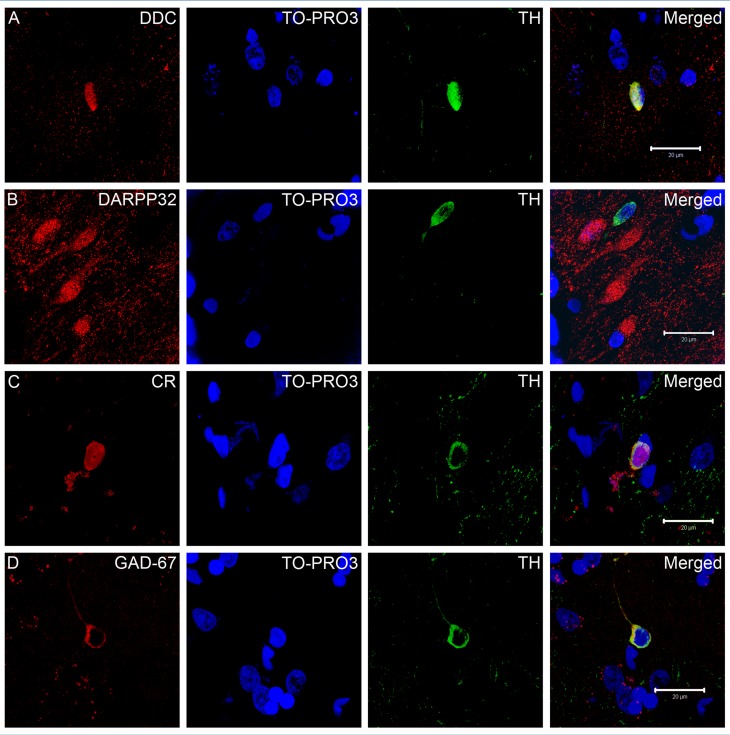
Double immunofluorescence images of the striatal TH-ir cells showing one TH-ir cell that also express dopa-decarboxilase (DDC) (A), absence of TH-ir cells immunolabeled with the dopamine and cyclic AMP-regulated phosphoprotein (DARPP-32) (B) TH-ir interneurons expressing calretinin (CR) (C) and glutamate decarboxylase (GAD67) (D). Scale bars 20 µm.

First of all, we searched for TH-ir striatal neurons that also expressed the marker of striatal projecting neurons DARPP-32 [23] and failed to indentify any TH/DARPP-32 double-labeled cell in any experimental group (Figure 5), which confirm the interneuron nature of this striatal cell population.

**Figure 6:**
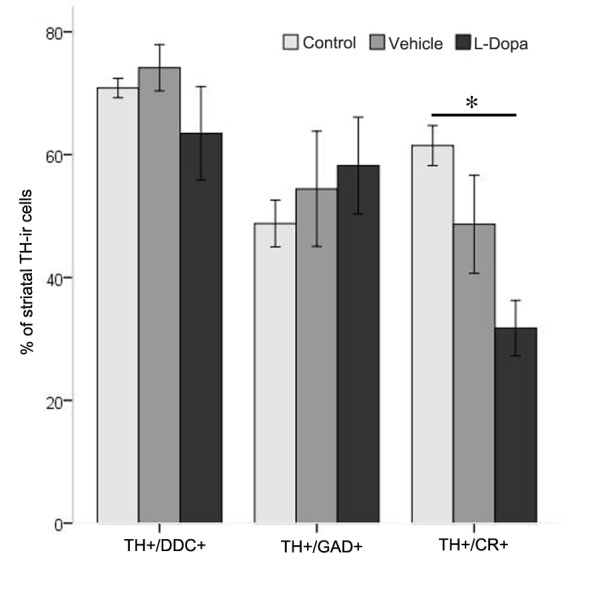
The graphic represents the percentage of TH-ir cells that co-localizes with glutamate decarboxylase (TH+/GAD67+), calretinin (TH+/CR+) and Dopa- decarboxylase (TH+/DDC+). Note the significant reduction of the double TH/CR immunoreactive cells in the L-Dopa group with respect to control group. * = p<0.05 using Tukey test following one way ANOVA repeated measures. Error bars represent SEM.

We found that in all experimental groups, striatal TH-ir cells expressed DDC and GAD67, but in different percentages. In the control group, about 70% of the TH-ir cells expressed DDC. This percentage of TH/DDC double-labeled cells was similar to that in the vehicle (74.15±3.76) and L-Dopa groups (63.47±7.61). In addition, the percentage of cells double labeled with TH and GAD67 was also identical in all experimental groups (48.78±3.80, 54.43±9.38 and 58.22±7.87 in control, vehicle and L-Dopa groups, respectively). As the majority of the striatal TH-ir cells are interneurons, we used calretinin (CR) to further characterize this cell population. Interestingly, in the L-Dopa-treated group the number of TH/CR double-labeled cells (31.75±4.50) was lower than that of the control group (61.47±3.26 p = 0.031). However, we did not find any difference when comparing control and vehicle groups (48.65±7.97, p = 0.15) and vehicle and L-Dopa groups (p = 0.46) (Figure 6).

**Figure 7:**
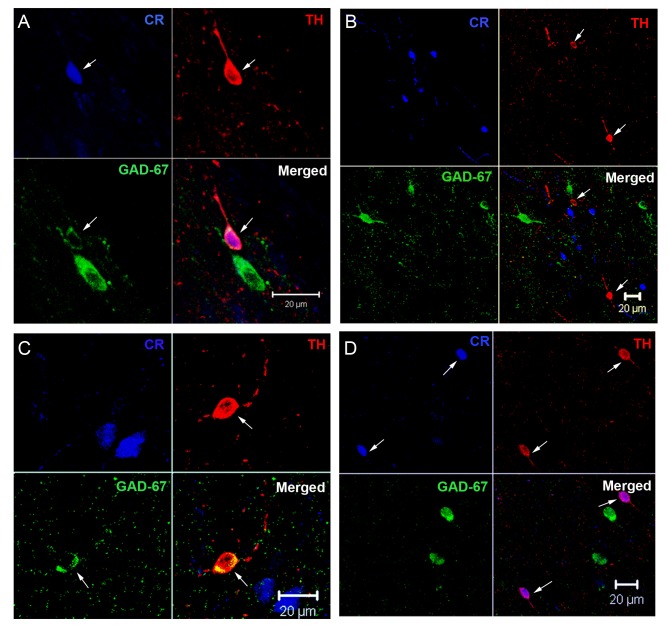
Triple immunofluorescence images showing the different 4 phenotypes of the TH-ir cells here described. A) TH-ir cell labeled with CR and GAD67, B) two TH-ir cells that do not express either CR or GAD67, C) TH-ir cell expressing GAD67 but not CR and D) two TH-ir cells expressing CR but not for GAD67. Scale bars 20 µm.

Since double IF labeling showed that the number of TH-ir cells expressing GAD67 and CR was about of 50% of the total cell population we further characterized the phenotype of these cells by performing triple TH/CR/GAD67 IF techniques. Interestingly, we were able to identify four different cell phenotypes 1) one type of TH-ir cells was positively labeled for GAD67 and CR, 2) the second type was immunoreactive for GAD67 but did not express CR, 3) the third phenotype of TH-ir neurons expressed CR but no GAD67 and 4) the fourth phenotype was formed by cells that do not express GAD67 or CR (Figure 7).

**Figure 8:**
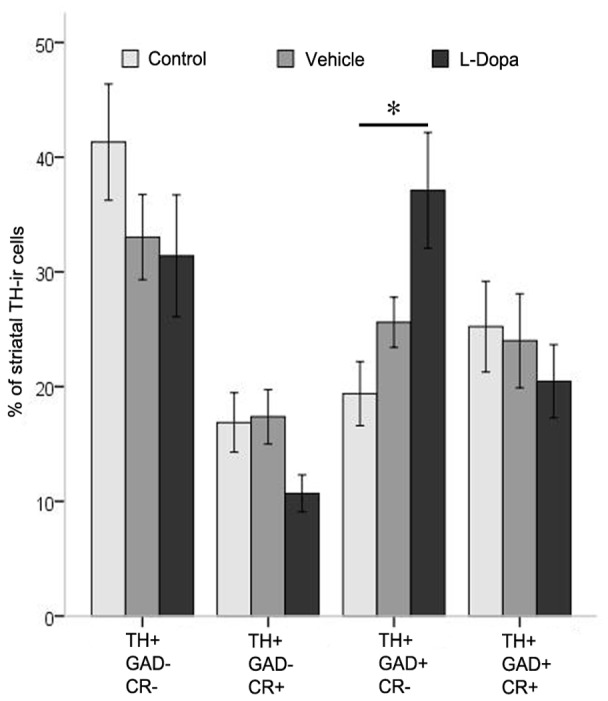
The graphic represents the results of triple immunofluorescence studies in the pre-commissural striatum of every experimental group and the percentage of TH-ir cells that co-localizes with glutamate decarboxylase (GAD67+) and calretinin (CR+). Note that phenotype TH+/GAD67+/CR- was significantly increased in the L-Dopa group with respect to control group (* = p<0.05 using Tukey test following one way ANOVA repeated measures). Error bars represent SEM.

All four phenotypes of TH-ir cells were present in the striatum of the three groups of animals but in different percentages. Thus in control and vehicle group the majority of TH-ir cells did not express GAD67 or CR (41% and 33% respectively), while in the L-Dopa group the more common phenotype of TH-ir cell was the one expressing GAD67 but not CR (37%). Statistical analysis showed that the proportion of TH-ir cells expressing GAD67 but not CR was significantly higher in the L-Dopa group than in the control group (p = 0.031) (Figure 8). In summary, the increased number of striatal TH-ir cells found in the L-Dopa group seems to preferentially affect the cell population expressing only GAD67.

## Discussion

We here confirm and extend previous studies showing that nigrostriatal degeneration induces a marked and significant increase in the number of striatal TH-ir cells in non-human primates. We also demonstrate that this increase is further enhanced by chronic L-Dopa exposure. In fact, we found that the striatum of parkinsonian monkeys chronically treated with L-Dopa exhibited a larger number of TH-ir cells than the vehicle and control animals particularly in the most anterior part of the pre-commissural striatum. Interestingly, this increase was observed after a washout period of 6 months. In contrast, chronic L-Dopa treatment does not alter the number of surviving nigral cells, confirming the lack of toxicity of L-Dopa in the primate nigral cell population. Finally, we also found that L-Dopa exposure provoked a phenotypic change in the striatal TH-ir cells with a significantly decreased number of the TH-ir cells expressing CR while the number of the TH/GAD67 double-labeled cells increased. This result indicates that levodopa modulates not only the number but also the phenotype of the striatal TH-ir neurons.

One of the most interesting findings in this study is the demonstration that L-Dopa seems to enhance the already increased number of TH-ir cells induced by chronic exposure to MPTP in non-human primates. However, our results appear not to be consistent with previous descriptions showing reduced rather than increased numbers of striatal TH-ir cells after L-Dopa administration in both humans and parkinsonian monkeys [11], [17]. Although a plausible explanation is not easy to find, differences in the method and in the experimental design followed might partially account for these discrepancies. For instance, in our study we used a washout period of 6 months to exclude any pharmacological effect of the drug which clearly differs from the report of PD patients who had received L-Dopa treatment until death and from the Huot’s study in which L-Dopa was given until sacrifice. In addition, differences in the extent of the nigrostriatal degeneration induced by MPTP might have also contributed. In fact, in the Huot’s study animals were given higher doses of MPTP (8-fold higher) and consequently they exhibited higher parkinsonian scores (60% of the maximum disability as compared with only 20% of maximum score of our animals). Moreover, they were treated with much higher doses of L-Dopa than our animals during shorter period of time. Another difference with previous studies might be found in the quantification method. For instance, in Huot et al. [17] report cell counting was performed using IF and found that L-Dopa decreased the number of striatal TH-ir cells while Palfi et al. [24] using stereology in brightfield, described an increase in the number of these cells after striatal GDNF delivery. However, our results are not completely in disagreement with the previous report since we cannot rule out the possibility that in our animals L-Dopa might have down-regulated the number of TH-ir cells while the treatment was active and then up-regulated it once L-Dopa was interrupted and the striatal dopamine content decreased.

By what mechanism could L-Dopa increase the number of the striatal TH-ir cells? Several hypotheses might be considered. One possibility is that the increased number of TH-ir cells merely reflects a compensatory striatal mechanism developed to convert into dopamine the exogenous L-Dopa [25]. The large proportion of the striatal TH-ir cells (70%) that express the DDC enzyme, and the increased striatal ^18^F-dopa uptake observed in the L-Dopa group as long as 6 months after concluding L-Dopa therapy, support this hypothesis [26]. The notorious distribution of the increased striatal TH-ir neurons in the more anterior part of the striatum and not in the motor putamen probably reflect an increased dopamine turnover in the surviving dopaminergic terminals as suggested by Porrit et al. [12].

Another explanation might be found in a possible trophic effect of dopaminergic denervation and L-Dopa therapy on the dopaminergic striatal cells [27]. Although the striatal levels of GDNF and other neurotrophic factors with proven effect on the survival, proliferation and differentiation of dopaminergic cells are very low, they markedly increased after nigrostriatal denervation, both in human and MPTP-monkeys [28]–[31]. On the other hand, striatal delivery of trophic factors by carotid body graft [29], neural stem cell implanted [32] or lentivirus [24], increase the number of striatal TH-ir cells indicating that these cells react not only to the dopamine depletion but also to the local levels of trophic factors. Interestingly, *in vivo* studies have demonstrated that L-Dopa is able to promote the synthesis and release of neurotrophic factors [33]. Consequently, the increased number of striatal TH-ir cells found in the L-Dopa animals might be the result of a neurotrophic effect promoted by the dopaminergic denervation and L-Dopa therapy acting synergistically. Accordingly, these two factors might operate together to promote the generation of new neurons from quiescent striatal progenitors.

The generation of new neurons in non-classical neurogenic areas such as the striatum and substantia nigra is controversial [34], [35]. In aged MPTP monkeys striatal TH-ir cells seem to be the result of a phenotypic change of preexisting GABAergic neurons. [10]. However it cannot rule out the possibility that in young monkeys and particularly in the initial state of parkinsonism, L-Dopa can promote the maturation of some striatal quiescent progenitor cells. Interestingly, we have recently found that the monkey striatum contains a population of striatal neurons that express the marker of neural progenitor Sox-2 and CR indicating they still retain a proliferative capacity and they might originate mature neurons under certain circumstances. Our findings that L-Dopa treatment increased the TH/GAD67 cell phenotype but decreased the number of cells expressing TH/CR suggest that L-Dopa might promote the maturation of some striatal CR cells to a dopaminergic phenotype. These findings do not indicate the generation of new neurons but would point to the possibility that L-Dopa can promote the maturation of some striatal quiescent progenitor cells. Several authors have questioned the functional significance of striatal TH-ir cells. Although the increased number of striatal TH-ir cells might have potential benefit in PD patients since they express DDC, they might also contribute to the appearance of some complication derived from long-term L-Dopa exposure. In fact we found that the majority of the striatal TH-ir cells are distributed within the associative and in less degree in the sensorimotor regions of the striatum [36], [37] indicating they might be involved in the generation of certain behavioural patterns, and particularly, in the induction of dyskinesias by creating aberrant circuits [38], [39]. Alternatively, these striatal TH-ir cells might also contribute to the release of dopamine in a non-regulated manner in some striatal areas [40], thus favoring the appearance of abnormal behaviors. In fact, it has been recently reported in mice that the scores of L-Dopa induced dyskinesias are closely associated with the number of striatal TH-ir cells [41].

In summary, we here report that L-Dopa administration produces a long-lasting increased number of dopaminergic cells along with a phenotypic shift of these cells to a more mature dopaminergic phenotype. However, further work is required to understand the functional implication of this phenomenon in the treatment of PD patient as well as to elucidate whether it might be beneficial or detrimental for PD patients.
